# Bacterial flagellin is a dominant, stable innate immune activator in the gastrointestinal contents of mice and rats

**DOI:** 10.1080/19490976.2023.2185031

**Published:** 2023-03-07

**Authors:** Matam Vijay-Kumar, Venugopal R. Bovilla, Beng San Yeoh, Rachel M. Golonka, Piu Saha, Bina Joe, Andrew T. Gewirtz

**Affiliations:** a UT Microbiome Consortium, Department of Physiology & Pharmacology, University of Toledo College of Medicine and Life Sciences, Toledo, OH, USA; b Center for Inflammation, Immunity and Infection, Institute for Biomedical Sciences, Georgia State University, Atlanta, GA, USA

**Keywords:** Gut microbiota, LPS, MyD88, toll-like Receptor-5, toll-like Receptor-4, cytokines, IL-10

## Abstract

Intestinal contents comprise the largest repository of immunogenic ligands of microbial origin. We undertook this study to assess the predominant microbe-associated molecular patterns (MAMPs) present therein and the receptors) that mediate the innate immune responses to them. Here, we demonstrated that intestinal contents from conventional, but not germ-free, mice and rats triggered robust innate immune responses *in vitro* and *in vivo*. Such immune responses were abrogated in the absence of either myeloid differentiation factor 88 (MyD88) or Toll-like receptor (TLR) 5, but not TLR4, suggesting that the stimuli was flagellin (*i.e*., protein subunit of flagella that drives bacterial motility). Accordingly, pre-treating intestinal extracts with proteinase, thereby degrading flagellin, was sufficient to block their ability to activate innate immune responses. Taken together, this work serves to underscore flagellin as a major, heat-stable and bioactive MAMP in the intestinal content that confers this milieu strong potential to trigger innate immune responses.

## Introduction

The mammalian intestines are residence for numerous microorganisms collectively known as the ‘gut microbiota’. It has been estimated that there are 10^11^ bacteria per gram of stool in the healthy colon^1^, thereby putting the colonic mucosa in very close proximity to the highly dense concentration of various microbial products. These microbe-associated molecular patterns (MAMPs) include ligands for pathogen recognition receptors (PRR) such as toll-like receptors (TLR) and nod-like receptors, which are in active dialog with intestinal epithelial cells (IEC) and mucosal immune cells in the lamina propria. Several mechanisms are in place to prevent or subdue activation of PRR by MAMPs, which include limiting the expression of PRR on the basolateral side of IEC thus separating them from their ligands in the gut lumen^2^, and secreting soluble forms of TLR that bind and sequester their ligands, thus preventing the ligands from inducing membrane-bound TLR signaling^3–5^. These protective mechanisms could be undermined by acute mucosal breaches and/or persistently compromised epithelial permeability (*alias* leaky gut), resulting in MAMPs translocating from the gut to other organs/tissues resulting in PRR activation and, consequently, inflammation.

The goal of this study was to better define the potential of the microbe-derived luminal contents to activate innate immunity when given broad access to extraintestinal host tissues. To this end, we administered mice with gastrointestinal extract contents via intraperitoneal injection to emulate scenarios where intestinal barrier was bypassed. Herein, we report that murine cecal content and fecal extracts indeed had high potential to activate innate immunity as reflected by induction of pro-inflammatory cytokine expression. However, such activation did not require TLR4; rather, such immune response was gut microbiota-dependent and driven by a heat-stable, protease-sensitive ligand whose signaling required the flagellin receptor, TLR5. These results suggest that flagellin (a protein and the principal component of bacterial flagella) is a dominant innate immune activator of gut microbiota, particularly in scenarios involving breach of the intestine.

## Material and methods

### Animals

Conventional WT, *Myd88*KO, *Tlr4*KO, *Tlr5*KO and *Il10*KO mice on C57BL/6 background, which were originally obtained from Jackson Laboratories, were housed and bred in the Department of Laboratory Animal Resources, University of Toledo College of Medicine and Life Sciences, Toledo, OH. The aforementioned genetically altered mice were bred with WT mice to generate their respective littermates. These mice were maintained on specific pathogen-free conditions and housed 4–5 per cage with Enrich-n’Pure paper bedding material, and provided *ad libitum* standard rodent chow diet from *LabDiets* (Cat #5001) and autoclaved water. Germ-free mice on C57BL/6 background from Taconic Biosciences (Rensselaer, NY) were bred and housed in the germ-free animal facility at Georgia State University. These mice were provided autoclaved *LabDiets* (Cat #5001) and water *ad libitum*, and were maintained in glove box isolators (Park Bioservices, Groveland, MA) and IsoCages (Techniplast Inc., Buguggiate, Italy). Germ-free and conventional male Sprague Dawley rats were obtained from Taconic Biosciences (Germantown, NY). These rats were provided *ad libitum* autoclaved normal chow diet (NIH31M) and water. All animal procedures, including breeding and weaning were performed in accordance with the Guide for the Care and Use of Laboratory Animals and were reviewed and approved by the Institutional Animal Care and Use Committee (IACUC) of the University of Toledo and Georgia State University.

### Processing of intestinal contents

Cecal contents and fecal pellets were aseptically collected from germ-free and conventional (*aka* germ-full) mice and rats and stored at −80°C. Samples were dried at 37°C overnight and pellets were homogenized (100 mg/ml) in sterile PBS using Precellys 24 Lysis and Tissue Homogenizer (Bertin Technologies). Homogenates were freeze-thawed one time, boiled for 10 min to lyse bacteria, and sonicated for 45 sec using Qsonica Sonicator to disrupt microbial cell wall components and dissociate the polymeric flagellum into flagellin monomers. We confirmed that intestinal extracts were completely free from live bacteria by plating extracts on nonselective agar plates. Finally, the samples were centrifuged at 12,000 rpm for 15 min and clear supernatants were collected and stored at −80°C until further use. In one experiment, we compared the immune responses induced by fully processed (as above) and semi-processed intestinal extracts, whereby the latter was prepared similarly but without the boiling and sonication steps.

### Protein estimation

Total protein content in fecal and cecal extracts was measured using Pierce^TM^ BCA Protein Assay Kit (Thermo Scientific, Rockford, IL) per manufacturer’s protocol.

### *In vitro* studies

Human model intestinal epithelial cell line (HT29) was grown using McCoy’s 5a medium supplemented with 10% FBS, 2 mM L-glutamine, 1% penicillin and streptomycin, and non-essential amino acids. Confluent HT29 cells were grown on plastic culture plates and on the day of stimulation, cells were washed twice with PBS and stimulated with 100 µg equivalent of fecal extracts (FE) or cecal extracts (CE) from either germ-free or conventional mice in serum-free DMEM for 5 h. HPLC-purified flagellin (FliC) from *Salmonella typhimurium* (SL3201, *fljB-*), prepared as described previously^6^, was used as positive control. Control wells were stimulated with PBS alone. Culture supernatants were collected and stored at −80°C until analyzed.

## *In vivo* administration of intestinal extracts

Eight-week-old male C57BL/6 mice (*aka* WT, *n* = 4–9) were administered 200 µg equivalent of intestinal extract protein intraperitoneally, *i.e*., 2.0 mg equivalent of cecal or fecal dry weight from either conventional or germ-free mice. Control mice received PBS. Mice were continuously monitored for food intake, moribundness and nestlet degradation for 2 h and then bled for serum collection. Body weights were also monitored after 24 h.

### Proteinase treatment

Conventional mice fecal extracts were digested with Proteinase K (0.5 mg/ml) overnight at 37ºC. Samples were boiled for 10 min to inactivate Proteinase K prior to administration in mice.

### Serum collection

Blood was drawn from non-anesthetized mice via mandibular vein bleeding, using a sterile lancet (Medipoint, Mineola, NY; Cat #98108–19570), and collected into BD microtainer tubes (Becton, Dickinson). After 30 min, tubes were centrifuged at 8,000 rpm for 10 min and hemolysis-free serum was collected and stored at −80°C until further analysis for cytokines.

### Cytokine analysis

Human IL-8, and mouse serum IL-6, KC, serum amyloid A (SAA) and lipocalin-2 (Lcn2) were quantitated with appropriate dilutions using Duoset ELISA kits from R&D Systems (Minneapolis, MN) according to the manufacturer’s protocol.

### Immunoblotting for flagellin in intestinal extracts

Mouse fecal and cecal extract (0.4 mg by dry weight or 40 µg protein equivalent) was processed in Laemmli buffer and subjected to 4–20% SDS-PAGE under reducing conditions. After transfer to polyvinylidene difluoride membrane, the blots were blocked with 5.0% nonfat milk for 1 h and probed overnight with affinity-purified rabbit anti-flagellin (1:500)^7^ for fecal extract immunoblot. For cecal extract immunoblot, the blots were probed overnight with serum from flagellin-immunized mice (1:500) for 1 h. After three washes, blots were incubated with anti-rabbit or anti-mouse horse horseradish peroxidase (1:1000) (ThermoFisher Scientific) for 1 h. After three washes, the blots were developed with an enhanced chemiluminescence (ECL) reagent (ThermoFisher Scientific).

### Statistical analysis

GraphPad Prism version 9.0 was used for statistical analysis. Two-tailed Student’s *t* test was used to compare between two groups, whereas one-way or two-way ANOVA with Tukey’s post-hoc test was used for studies with >2 groups with one or two variables, respectively. Statistically significant values were represented as *p* ≤ 0.05 (*), *p* ≤ 0.01 (**), and *p* ≤ 0.001 (***). All data were expressed as mean ± SEM.

## Results

### In vivo administration of intestinal extracts robustly induced pro-inflammatory cytokines

The potential of intestinal contents to trigger inflammatory responses warrants identification of the predominant MAMPs present therein, as well as the receptor(s) that mediate those responses. During the preparation of fecal (FE) and cecal (CE) extracts from conventional (Conv) and germ-free (GF) mice, we initially expected that the presence of gut microbiota (encompassing bacteria, fungi, and viruses) would increase the total protein content in the extract preparation. The protein contents of Conv FE and CE, however, were comparable to their GF counterparts, where total protein is approximately 10% w/w of dry weight of feces and cecal content, respectively (Figure S1). To test whether the intestinal extracts can activate immune responses *in vitro*, we challenged a human model IEC with 100 µg of protein equivalent of FE and CE. Intriguingly, FE and CE from Conv mice induced IL-8 secretion from human IEC (Figure S2). Such IL-8 response was not observed in IEC treated with either FE or CE from GF mice, affirming that this chemokine response was potentiated by a bacterial product(s).

**Figure 1.**
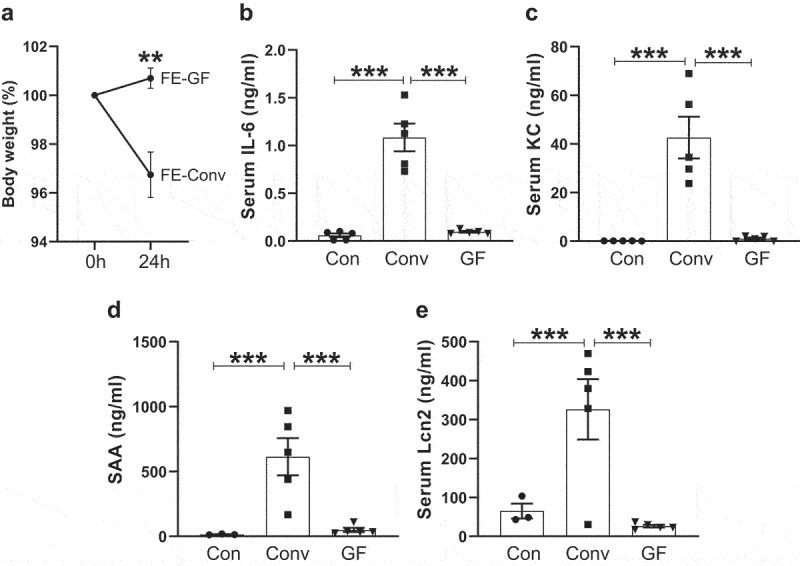
*In vivo* administration of intestinal extracts from conventional but not germ-free mice induced robust pro-inflammatory cytokine and chemokine responses. Eight-week-old female C57BL/6 mice (*n* = 5) were administered FE (2.0 mg equivalent of dry feces weight; *i.P*.) from either conventional (Conv) mice or germ-free (GF) mice. Control mice were given sterile PBS. **(a)** Body weight (%) at 0 and 24 h post-treatment. After 2 h post-treatment, mice were bled and hemolysis-free sera were analyzed for **(b)** IL-6, **(c)** KC, **(d)** serum amyloid a (SAA) and **(e)** Lipocalin-2 (Lcn2). Results were expressed as mean ± SEM. Statistical significance calculated using two-tailed Student’s *t* test for **(a)** and one-way ANOVA with Tukey’s post-hoc test for **(b-e)**. **p < 0.01, ***p < 0.001.

Next, to test whether the intestinal extracts can activate immune responses *in vivo*, we intraperitoneally administered either PBS or 0.2 mg of FE protein (2.0 mg dry weight equivalent of FE) to wild-type (WT) C57BL/6 mice. After 15 min post-administration of FE from Conv mice, the recipient mice exhibited inactivity, distress and did not engage in nestlet shredding. These symptoms were absent in mice given PBS or FE from GF mice. Intriguingly, mice receiving Conv-FE, but not GF-FE, exhibited a modest loss in body weight 24 h post-treatment (Figure 1a), suggesting that the former may have underwent a cytokine storm following exposure to MAMPs. Analysis of sera collected 2 h post-challenge of Conv-FE showed striking elevations in IL-6 and KC; such response was absent in mice given either PBS or FE from GF mice (Figure 1b,c). Similarly, FE from Conv mice also induced substantial levels of acute phase proteins, which serve as general inflammatory markers, such as serum amyloid A (SAA) and lipocalin-2 (Lcn2); however, SAA and Lcn2 were not induced by FE from GF mice (Figure 1d,e). Interestingly, serum IL-1β and tumor necrosis factor alpha (TNFα) were not detectable (data not shown) following FE treatment, suggesting that immune response to intestinal extracts is rather specific to IL-6 and KC rather than a broad cytokine storm response. Results from FE treatment can be recapitulated with CE, whereby only CE from Conv, but not GF, mice induced IL-6 and KC response (Figure S3a,b). Moreover, the pro-inflammatory response can also be recapitulated using FE from rats instead of mice (Figure S4a-d), indicating that the response is conserved irrespective of the source of intestinal extracts.

### Induction of innate immune response by intestinal extracts is MyD88 dependent

**Figure 2.**
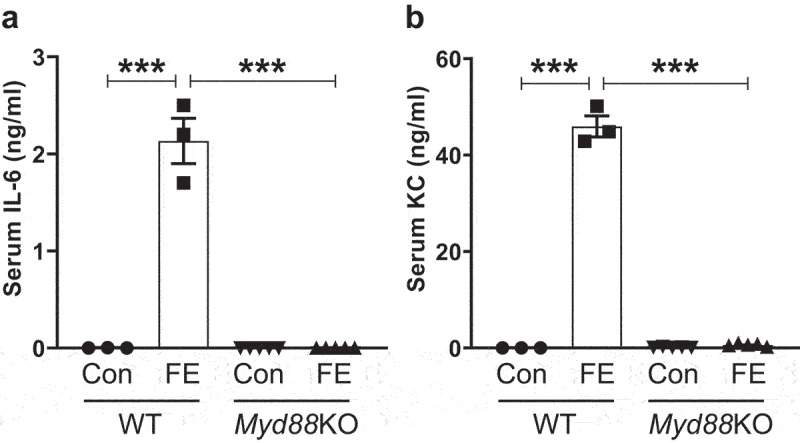
Intestinal extracts from conventional mice failed to induce pro-inflammatory cytokine response in Myd88 deficient mice. Eight-week-old male MyD88-deficient (*Myd88*KO) mice (*n* = 5) and their WT littermates (*n* = 3) were administered FE (2.0 mg equivalent of dry feces weight; *i.P*.) from conventional mice. Control (Con) mice were given sterile PBS. After 2 h, mice were bled, and hemolysis-free sera were analyzed for **(a)** IL-6 and **(b)** KC. Results were expressed as mean ± SEM. Statistical significance calculated using two-way ANOVA with Tukey’s post-hoc test. ***p < 0.01.

Next, we asked whether the induction of pro-inflammatory cytokines by FE is dependent on myeloid differentiation factor 88 (MyD88), a global adapter protein required for all TLR signaling, except TLR3. Therefore, we intraperitoneally administered 0.2 mg of FE protein (2.0 mg fecal dry weight equivalent) from Conv mice to either WT or MyD88-deficient (*Myd88*KO) mice and analyzed their serum cytokines after 2 h. Remarkably, WT mice responded robustly to FE from Conv mice by upregulating serum IL-6 and KC (Figure 2a,b). In contrast, *Myd88*KO mice were completely refractory to FE from Conv mice (Figure 2a,b), suggesting MyD88 is absolutely required for FE-induced innate immune responses. Since MyD88 signaling is known to be inhibited by interleukin-10 (IL-10)^8^, we next asked whether loss of IL-10 in mice can augment their innate immune response against FE. Indeed, Conv-FE induced IL-6 and KC more robustly in IL-10 deficient mice (*Il10*KO) than WT mice (Figure S5a,b). Conversely, GF-FE failed to induce such innate immune response (Figure S5a,b). Thus, these results collectively suggest that the PRRs which are responding to intestinal extracts require MyD88.

### Innate immune response induced by intestinal extracts is TLR5 dependent

**Figure 3.**
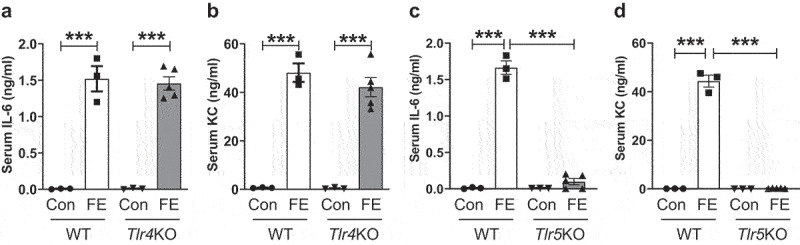
Intestinal extracts-induced pro-inflammatory response is TLR5-dependent. Eight-week-old male WT, TLR4-deficient (*Tlr4*KO) or TLR5-deficient (*Tlr5*KO) mice (*n* = 3–5) were administered FE (2.0 mg equivalent of dry feces weight; *i.P*.) from conventional mice. Control (Con) mice were given sterile PBS. After 2 h, mice were bled, and hemolysis-free sera were analyzed for **(a&b)** IL-6 and KC in WT and *Tlr4*KO mice and **(c&d)** IL-6 and KC in WT and *Tlr5*KO mice. Results were expressed as mean ± SEM. Statistical significance calculated using two-way ANOVA with Tukey’s post-hoc test. ***p < 0.001.

The preparation of FE involves boiling and sonication procedures, which not only kill any live bacteria and other microorganisms but also facilitate lysis, disruption, and release of microbial products. We reasoned that the MAMP present in FE are heat-stable and can remain intact and bioactive. We first speculated that the TLR4 ligand, *i.e*., LPS, which is heat-resistant, could be accountable for the robust *in vivo* innate immune responses toward FE. Therefore, we intraperitoneally administered FE from Conv (0.2 mg protein equivalent) to either WT or TLR4-deficient (*Tlr4*KO) mice and analyzed their serum cytokines after 2 h. Intriguingly, the induction of serum IL-6 and KC toward FE from Conv mice was completely intact in *Tlr4*KO mice as in WT mice (Figure 3a,b). This surprising outcome suggests that the immunogenicity of FE was independent of TLR4 and thus led us to next examine whether it was dependent on TLR5 whose cognate ligand, *i.e.*, flagellin, is also heat-stable. Remarkably, the induction of serum IL-6 and KC was completely muted in *Tlr5*KO mice (Figure 3c,d). Thus, this underscores that the innate immune response to intestinal extracts requires TLR5.

### Immunogenicity of intestinal extracts can be attributable to flagellin

**Figure 4.**
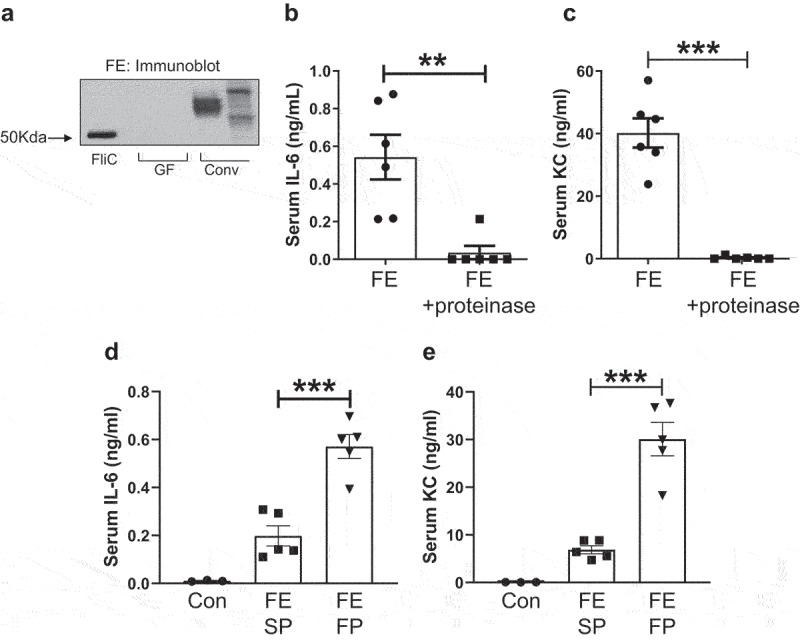
The immunogenicity of fecal extract was attributable to flagellin, which can be dampened by proteinase treatment or heightened by heating and depolymerization. (a) Flagellin immunoblot showing the absence and presence of flagellin in germ-free (GF) and conventional (Conv) mouse feces, respectively. (**b&c**) FE from Conv mice were digested with or without Proteinase K (1 mg/ml) overnight at 37ºC. Samples were boiled for 10 min to inactivate Proteinase K prior to administering to eight-week-old male mice (*n* = 6) at a dose of 200 µg protein equivalent (*i.P*.). After 2 h, mice were bled and hemolysis-free sera were analyzed for **(b)** IL-6 and **(c)** KC. (**d&e**) in a separate experiment, eight-week-old female mice (*n* = 3–5) were challenged with FE (2.0 mg equivalent of dry feces weight; *i.P*.) that were either semi-processed (SP: without boiling or sonication) or fully processed (FP: with boiling and sonication); see Methods section for details. After 2 h, mice were bled and hemolysis-free sera were analyzed for **(d)** IL-6 and **(e)** KC. Results were expressed as mean ± SEM. Statistical significance calculated using two-tailed Student’s *t* test for **(b-c)** and one-way ANOVA with Tukey’s post-hoc test for **(d-e)**. **p < 0.01, ***p < 0.001.

The requirement of MyD88 and TLR5, but not TLR4, in the cytokine response to FE suggests that the specific MAMP involved could be bacterial flagellin. Accordingly, we performed anti-flagellin immunoblotting of FE and CE alongside a HPLC-purified flagellin monomer, FliC isolated from *Salmonella typhimurium* as positive control (Figure 4a). The multiple bands seen in the immunoblot of FE from Conv mice reflect a diverse flagellin monomers and/or dimers of varying molecular weight expressed by various bacteria (Figure 4a). Positive bands, *albeit* less distinct, were also observed in the immunoblot of CE from Conv mice (Figure S6). More importantly, the absence of bands in the negative control, *i.e*., FE and CE from GF mice, substantiated our deduction that the positive bands seen in Conv intestinal extracts are flagellin.

If the immunogenic ligand in intestinal extracts is flagellin, which is a protein, we reasoned that degrading it with proteinase could blunt its ability to induce cytokine response. Accordingly, we pre-treated FE with proteinase K followed by heat-inactivation of the proteinase prior to *in vivo* administration. As anticipated, protein-digested FE failed to induce any elevation in serum KC or IL-6 (Figure 4b,c), thus implicating the immunogenic ligand in intestinal extracts is flagellin by virtue of it being the major, if not the only, well-characterized proteinaceous TLR ligand. Interestingly, semi-processed FE, *i.e*., not subjected to boiling and sonication, induced substantially reduced levels of KC and IL-6 by 82.0 and 64.0%, respectively, in *in vivo*, when compared to fully processed FE (Figure 4d,e). This finding is consistent with the notion that native flagellin polymers are poor agonists of TLR5 due to their recognition sites being buried in filamentous structure, whereas monomeric flagellin that are released upon drastic treatment are potent in activating TLR5^9^. Taken together, our findings pinpoint flagellin as the immunogenic factor in intestinal extracts that elicit KC and IL-6 through activation of TLR5.

## Discussion

The mammalian intestine harbors an enormous microbial biomass enriched with MAMPs that could elicit inflammation. It is estimated that the amount of LPS in the gut can reach to over 1.0 ng/ml^10^, whereby an *E. coli* contains 10^6^ LPS molecules per cell^11^. Its ubiquitous presence in the gut as a bacterial cell wall component as well as its heat stability (*i.e*., not easily degradable unlike DNA or RNA) have led LPS to be considered a major immunogenic MAMP in the gut and as a factor contributing to the pathogenesis of sepsis. However, efforts thus far in developing anti-LPS-based therapeutics for treating sepsis have not met with any clinical success^12–14^. Perhaps, these outcomes should not be surprising if one considers that the total LPS pool in the intestine is mostly comprised of non-immunogenic LPS variants derived from *Bacteroidales*, which belong to the phylum Bacteroidetes^15,16^. Unlike LPS derived from *E. coli* (whose abundance in the gut is rather low under healthy conditions), which is known to be pro-inflammatory, *Bacteroidales* LPS is notable for their inhibitory effects on innate immune signaling and in facilitating endotoxin tolerance^16^. This assertion was further tested in a study, which reported that the human gut-derived total LPS, as a whole, favors the inhibition of TLR signaling and exerts potent anti-inflammatory effects to promote mucosal tolerance toward LPS-expressing bacteria^15^. Such lack of immunogenicity displayed by gut microbiota derived LPS thus raises question on which MAMP(s) is responsible for inducing pro-inflammatory cytokine responses during conditions of leaky gut and/or sepsis.

It is perhaps not surprising that the intestinal contents are highly immunogenic and potent in eliciting robust IL-6 and KC responses *in vitro* and *in vivo*. However, the requirement of MyD88 and TLR5, but not TLR4, in such responses was rather unexpected and, moreover, it suggests that the instigative MAMP in the gut could be flagellin instead of LPS. This notion is supported, firstly, by the loss of cytokine responses upon treating FE with proteinase. Secondly, FE was notably more potent in inducing innate immune responses following boiling and sonication. These two observations align with the notion that flagellin is a proteinaceous TLR ligand and that depolymerizing the native flagellum polymer by boiling, thus releasing its monomeric form has been shown to increase its potency in activating TLR5^9^. We initially expected that systemic exposure to intestinal extracts would trigger a robust LPS-induced signature or ‘cytokine storm’ with copius amounts of circulating TNFα and IL-1β resulting in septic shock-like syndrome. However, lack of such septic-like response in intestinal extract given mice suggests that LPS might be (i) detoxified via intestinal alkaline phosphatase, (ii) luminal bile acids-bound LPS may be inactive or not accessible to its co-receptors and thus TLR4 or (iii) that gut-derived total LPS are largely non-immunogenic and thus unlikely to contribute to the immunogenicity of intestinal contents^15,16^.

A healthy human microbiome has been predicted to encode for more than 5,000 diverse flagellins, *albeit* flagellin-producing bacteria are mostly members of the phyla Firmicutes and Proteobacteria^17^, such as *Lachnospiraceae*, *Roseburia*, *Eubacterium*, and *Enterobacteriaceae*^18^. When compared to other known TLR ligands, flagellin is an exception as a well-characterized, proteinaceous ligand capable of activating both innate and adaptive immune responses. Flagellin is specifically potent in eliciting pro-inflammatory cytokines and chemokines, predominantly IL-6 and KC (the mouse homologue of human IL-8). Aside from activating innate immunity, flagellin also acts as an antigen to potentiate the generation of flagellin-specific antibodies predominantly IgG and IgA. The extent to which immune activation by flagellin could impact host health, however, remains poorly understood. On one hand, elevated titer of anti-flagellin IgG has been associated with Crohn’s disease^19,20^ and, moreover, flagellin-specific CD4^+^ cells have been reported to induce colitis in SCID mice^19^. The TLR5-dependent pro-inflammatory response exerted by pancreatic islets against flagellin has also been associated with beta-cell dysfunction and a loss in glycemic control^21^. Yet, on the other hand, our group and others have shown that the innate immune response elicited against *Salmonella* flagellin, when triggered in a prophylactic manner, could confer a broad spectrum protection against a chemical colitogen, radiation toxicity and viral and bacterial infections^22–26^. Such dual roles in which flagellin demote and promote health certainly warrants further investigation and, considering the potency of flagellin in activating innate immunity, perhaps the assessment on fecal/cecal reactivity of flagellin as tested herein can be further harnessed as a simple and cost-effective means to assess the status of gut microbiota eubiosis or dysbiosis.

In conclusion, we have obtained multiple evidences that indicate flagellin, a TLR5 *bona fide* ligand, as the dominant MAMP in intestinal contents. This includes our observation on the (i) no induction of innate immune responses *in vitro* or *in vivo* by germ-free intestinal extracts, (ii) loss of bioactivity upon protease treatment, (iii) the requirement for harsh treatment (boiling and sonication) to dissociate polymeric flagellum to flagellin monomers, (iv) heat stability, and (v) complete loss of bioactivity in *Tlr5*KO mice. One limitation in our study approach was that intestinal contents were prepared as extracts *ex vivo* and therefore may not retain their native composition. This point notwithstanding, the fecal and cecal samples were immediately frozen at −80ºC degrees and the subsequent drying occurred relatively rapidly, thus not providing much window for bacterial growth/change in microbiota composition. That said, we acknowledge that we cannot entirely eliminate the possibility that some growth/change did occur. Yet, irrespective of whether any growth/changes during the drying process occurred, our central conclusion was still applicable, i.e., the induction of immune responses by intestinal extracts was TLR4-independent, but TLR5-dependent. *In lieu* of LPS, identification of specific bacterial and archaeal flagellins responsible for the innate immune responses against intestinal extracts is certainly warranted and awaits further investigation.

## Supplementary Material

Supplemental Material

## Data Availability

The data that support the findings of this study are available from the corresponding authors upon reasonable request.
