# Supplementary material for: Bacterial flagellin is a dominant, stable innate immune activator in the gastrointestinal contents of mice and rats
Source: Gut Microbes. 2023 Mar 7;15(1):2185031. doi: 10.1080/19490976.2023.2185031 (PMC10012918; doi:10.1080/19490976.2023.2185031)

Supplementary Fig. S1

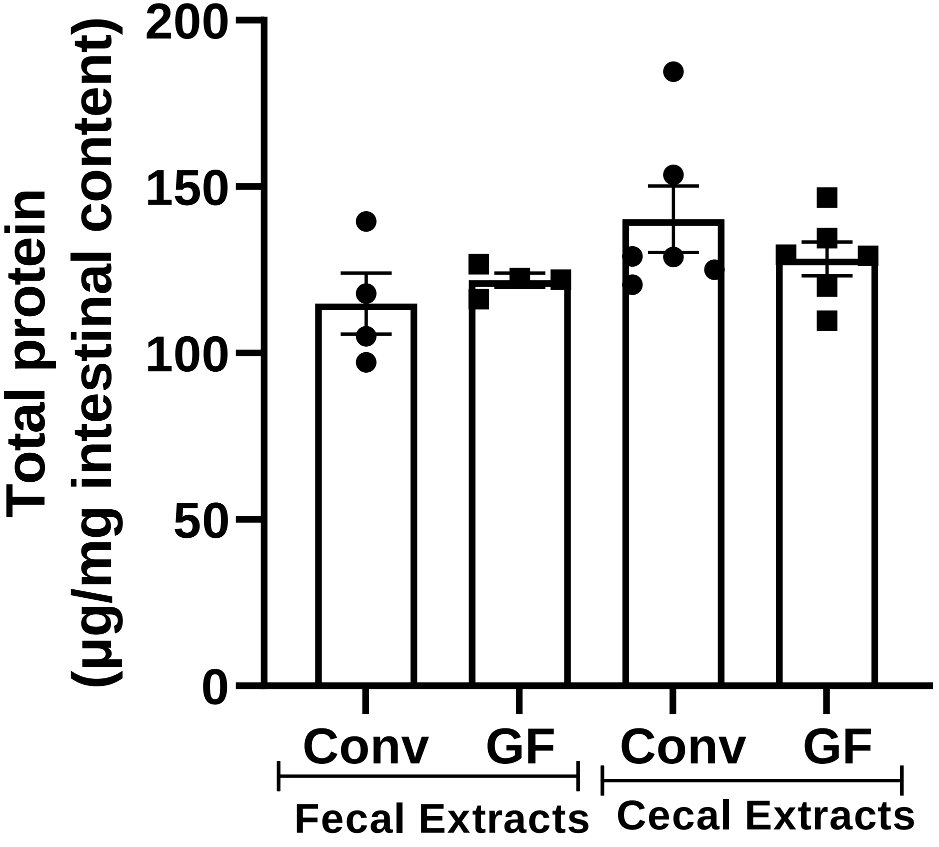

Supplementary Fig. S2

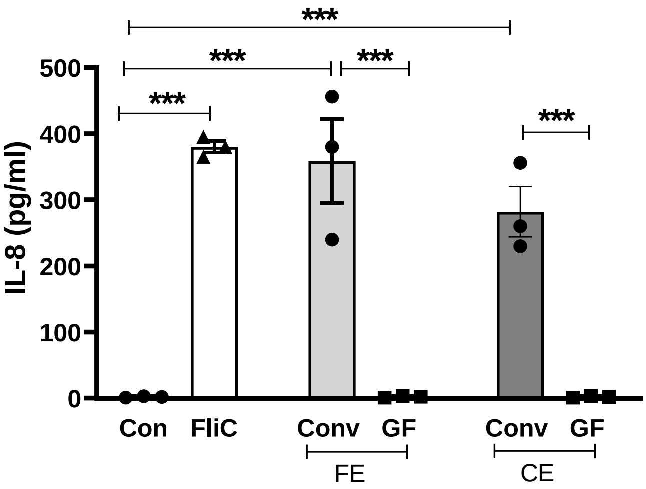

Supplementary Fig. S3

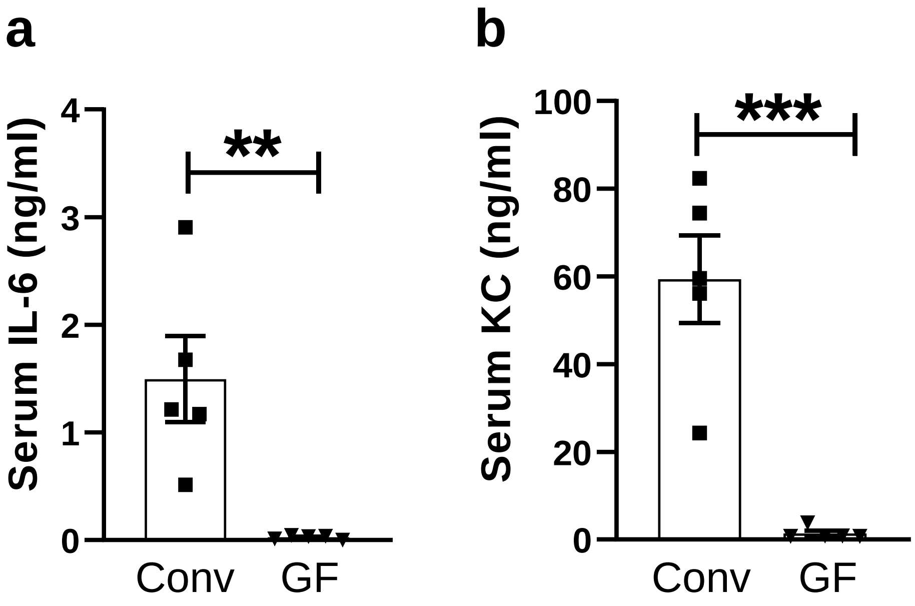

Supplementary Fig. S4

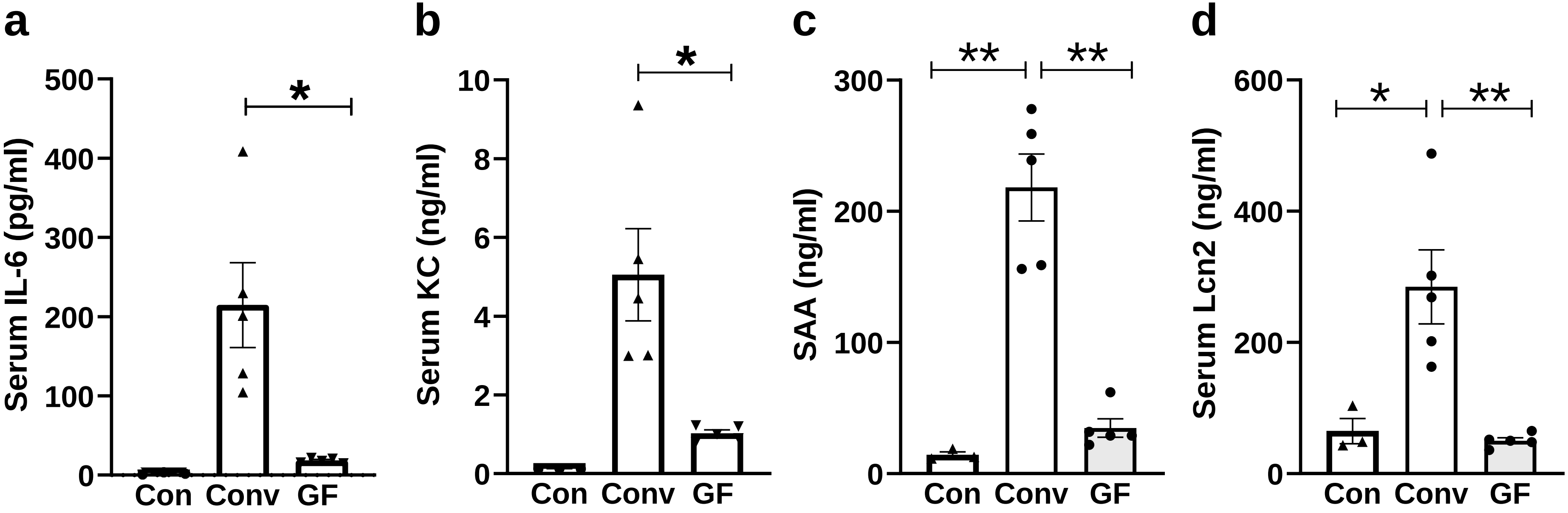

Supplementary Fig. S5

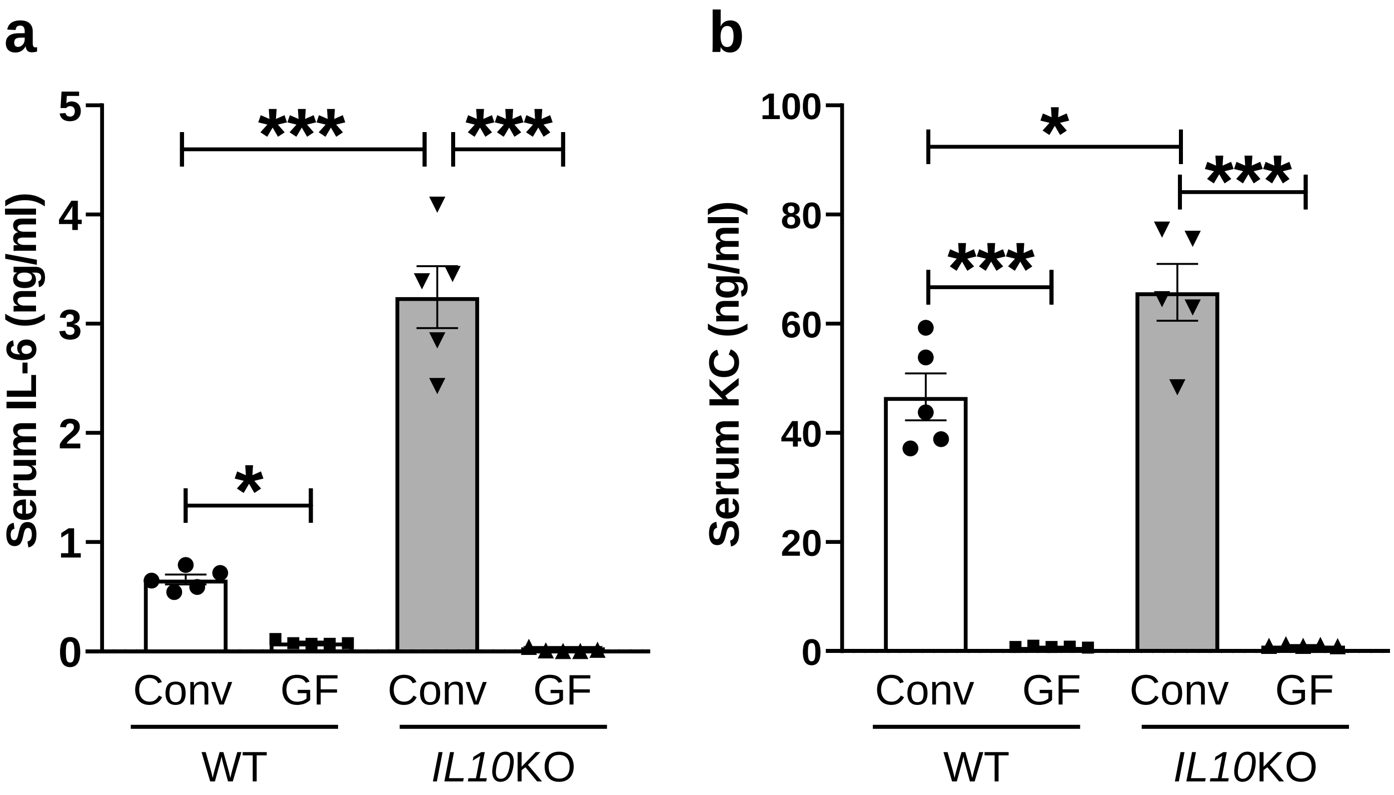

Supplementary Fig. S6

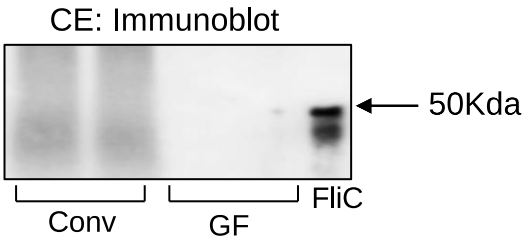

Supplement: Supplemental Material [file KGMI_A_2185031_SM7603.pdf]
